# Simultaneous Pancreas Kidney Transplantation Improves Cardiovascular Autonomic Neuropathy with Improved Valsalva Ratio as the Most Precocious Test

**DOI:** 10.1155/2020/7574628

**Published:** 2020-04-06

**Authors:** María Argente-Pla, Antonia Pérez-Lázaro, Antonio Martinez-Millana, María Isabel Del Olmo-García, Jordi Espí-Reig, Isabel Beneyto-Castello, Rafael López-Andújar, Juan Francisco Merino-Torres

**Affiliations:** ^1^Endocrinology and Nutrition Department, Hospital Universitario i Politécnico La Fe, Valencia, Spain; ^2^Mixed Research Unit of Endocrinology, Nutrition and Dietetics, La Fe Health Research Institute, Valencia, Spain; ^3^ITACA, Universitat Politècnica de València, Camino de Vera s/n, 46022 Valencia, Spain; ^4^Nephrology Department, Hospital Universitario i Politécnico La Fe, Valencia, Spain; ^5^Hepato-pancreato-biliary (HPB) Surgery and Transplant Unit, Hospital Universitario i Politécnico La Fe, Valencia, Spain; ^6^Medicine Department, Universitat de Valencia, Spain

## Abstract

**Background:**

Simultaneous pancreas-kidney (SPK) transplantation is a proven option of treatment for patients with type 1 diabetes mellitus (T1DM) and related end-stage renal disease. There is discrepancy between the results of different studies about the impact of prolonged normalization of glucose metabolism achieved by SPK on the course of diabetic complications including severe forms of diabetic neuropathy. The objective of the study was to evaluate the prevalence of cardiovascular autonomic neuropathy (CAN) in patients undergoing SPK transplantation and its evolution 10 years after transplantation.

**Methods:**

Prospective study of 81 patients transplanted in a single center from year 2002 to 2015. Autonomic function was assessed using cardiovascular autonomic reflex tests (CARTs). CARTs were made before SPK transplantation and during the follow-up. Evolution of tests after SPK transplantation was evaluated by contrasting hypotheses (paired tests). Multiple testing was adjusted with the Benjamini-Hochberg procedure with a false discovery rate of 10%.

**Results:**

48 males and 33 females, mean age 37.4 ± 5.7 years, mean BMI 24.0 ± 3.4 kg/m^2^, and mean duration of diabetes 25.5 ± 6.5 years, received SPK transplantation. Ten years after SPK transplantation, 56 patients re tained the pancreatic graft (42 of them with normofunctioning pancreas and 14 with low doses of insulin therapy). These 42 patients were selected for the autonomic study. Before transplant procedure, all CART results were abnormal. After SPK transplantation, paired test analysis showed an improvement of systolic blood pressure (SBP) response to orthostasis at the 5^th^ year after SPK (*p* = 0.03), as well as improvement of the Valsalva ratio at the 3^rd^ (*p* < 0.001) and 5^th^ (*p* = 0.001) year after SPK. After correcting for the false discovery rate, all the variables of autonomic study reached significance at different time points.

**Conclusions:**

Prevalence of CAN in patients who are candidates for SPK transplantation is high and is generally advanced. SPK transplantation improves CAN with improved Valsalva ratio as the most precocious test.

## 1. Introduction

Diabetic neuropathy is a common microvascular complication of long-term diabetes mellitus (DM). Its etiopathogenesis is multifactorial, but hyperglycemia seems to be the primary cause: increased accumulation of sorbitol and fructose in the nerves and decreased myoinositol and Na/K ATPase activity. Other mechanisms such as hypoglycemia [1], immunological mechanisms (antiganglioside antibodies), antiphospholipid antibodies, or microvascular insufficiency/ischemia are also involved in its etiopathogenesis [2].

Diabetic autonomic neuropathy (DAN) is a poorly studied complication of DM, despite its frequency and the significant negative impact it has on the survival and quality of life of diabetic patients [3]. DAN usually appears in long-standing diabetic patients, with poor metabolic control and is associated with the presence of other complications [4].

Cardiovascular autonomic neuropathy (CAN) is the most frequent autonomic complication in DM. CAN is defined as an alteration of the cardiovascular response to various visceral reflexes and is associated with a significant amount of the morbidity and mortality of diabetes. The literature shows a varying CAN prevalence depending on the criteria used for diagnosis, the population studied, and the time of DM evolution [5–8]: autonomic involvement varies in a range from 2 to 95% in type 1 DM (T1DM) and 25 to 75% in type 2 DM [5, 6] and up to around 90% in patients with a long-standing type 1 DM who are candidates for a pancreas transplantation [7].

CAN usually manifests itself subclinically, characterized by the alteration of cardiovascular reflexes such as resting tachycardia, or clinically, less frequently, and generally manifested as exercise intolerance, orthostatic hypotension, silent myocardial infarction, and intraoperative cardiovascular liability [5, 9–12]. Orthostatic hypotension is the most incapacitating manifestation of autonomic failure and is a consequence of reduced vasoconstriction of the splanchnic and other peripheral vascular beds due to efferent sympathetic vasomotor denervation [1]. As the symptoms and signs for CAN are nonspecific, early diagnosis and follow-up require special techniques based on heart rate (HR) variability. Cardiovascular autonomic reflex tests (CARTs) or “Ewing test” are considered the “Gold standard” [10–13]. All T1DM patients should be assessed for diabetic peripheral neuropathy starting 5 years after diagnosis and at least annually thereafter [14].

On the other hand, pancreas transplantation is a proven option of treatment for patients with T1DM and related end-stage renal disease, who are candidates for kidney transplantation. Three types of whole pancreas transplantation are described: simultaneous pancreas kidney transplantation (SPK), pancreas after kidney transplantation (PAK), and pancreas transplantation alone (PTA) [15]. Before pancreas transplantation, these patients usually present other microvascular complications derived from their DM, generally in an advanced stage such as CAN [7]. Successful pancreas transplantation achieves long-term normoglycemia and allows the assessment of the effect of prolonged normalization of the glucose metabolism on the course of diabetic complications including severe forms of diabetic neuropathy [16].

However, the data currently available on the improvement of neuropathy of pancreas-kidney transplant patients is limited, and there are doubts about the chronological evolution after transplantation [16–19]. To the best of our knowledge, no study has so far indicated which CART is first modified after pancreas transplantation.

This report describes the results of clinical examination and autonomic function tests in patients with T1DM before and up to 10 years after successful SPK transplantation.

## 2. Methods

### 2.1. Patients

This is a prospective study of 81 patients with T1DM (48 males and 33 females, mean age 37.4 ± 5.7 years, mean BMI 24.0 ± 3.4 kg/m^2^, and mean duration of diabetes 25.5 ± 6.5 years) who received SPK transplantation, at La Fe University Hospital in Valencia (Spain), between 2002 and 2015.

The inclusion criteria for SPK transplantation were T1DM patients (C − peptide < 0.5 ng/mL), end-stage renal disease (glomerular filtration rate < 20 mL/min/1.73 m^2^), below 50 years of age, with absence of severe psychiatric or psychological disorders and ability to understand what a pancreas transplantation entails in relation to postoperative collaboration, and complications that may arise in the follow-up of treatment.

**Figure 1:**
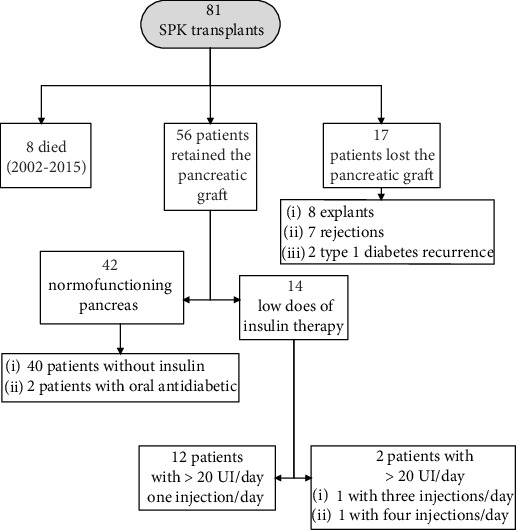
SPK transplant evolution.

After SPK transplantation, the maintenance of the function of the pancreatic graft and renal graft was defined as the achievement of complete independence from insulin and dialysis, respectively. Renal graft loss was considered as the need for dialysis after transplantation. Pancreas graft loss was considered; when after the transplantation, the graft had a partial function that required the use of exogenous insulin; when it presented a rejection, T1DM recurrence or a pancreatectomy was performed due to surgical complications (Figure 1). After SPK transplantation, 4 patients required nephrectomy due to surgical complications; these patients also suffered pancreatic function loss requiring exogenous insulin and, therefore, were not selected for the study.

After SPK transplantation, 56 patients retained pancreatic graft: 14 of them required low doses of insulin therapy, 0.25 UI/kg/day, and 42 presented normofunctioning pancreas without exogenous insulin (Figure 1).

Demographical, clinical, and biochemical data were collected in these 42 patients.

### 2.2. Transplantation Procedure

Our transplantation procedure has been previously described [20].

#### 2.2.1. Surgical Technique

All pancreas transplantations were performed by the same surgical team. All pancreatic and kidney grafts were procured from deceased donors, and both were placed extraperitoneally.

#### 2.2.2. Immunosuppression

Antithymocyte globulin or basiliximab were used for induction immunosuppression therapy. As maintenance immunosuppression therapy, patients are currently treated with a combination therapy consisting of tacrolimus, mycophenolate mofetil or sirolimus, and prednisone.

### 2.3. Laboratory Methods

Biochemical parameters were evaluated with different laboratory techniques. Serum glucose, urea, creatinine, total cholesterol, high-density lipoprotein cholesterol (HDL-c), and triglycerides levels were measured by enzymatic and colorimetric techniques (Cobas c701; ROCHE/HITACHI).

C-peptide serum levels were measured using an electrochemiluminescence immunoassay (Cobas c602).

Glycated hemoglobin (HbA1c) was measured using high-performance liquid chromatography (HPLC) (Horiba Tosoh G11).

The glomerular filtration rate (GFR) was calculated from serum creatinine levels using the Chronic Kidney Disease Epidemiology Collaboration (CKD-EPI) equation.

Finally, estimated low-density lipoprotein cholesterol (LDL-c) was calculated using Friedewald's equation.

### 2.4. Neurological Evaluation

Neurological evaluation was made before SPK transplantation and during follow-up: after 3, 5, 7, and 10 years. Autonomic function was assessed using CARTs that measure systolic blood pressure (SBP) responses to standing up and HR responses to the Valsalva maneuver and to deep breathing [21–23]. All CARTs were performed after an appropriate wash out (24 hours) of the antihypertensive treatment.

Expiration/inspiration (*E*/*I*) or deep breathing ratio assesses HR variability induced by a series of deep breathing cycles. This test is based on the fact that the HR decreases during expiration and increases during inspiration. With the patient at rest and supine for at least 10 minutes, HR is monitored by electrocardiogram (ECG) while the patient performs 6 deep breathing cycles, one breath every 10 seconds. The quotient between the longest R-R during expiration and the shortest R-R during the inspiration phase is performed in 3 of the 6 cycles, with the final result being the average of the 3 determinations. A difference of >15 beats per minute (bpm) are considered normal and <10 bpm is abnormal. The lowest normal value for *E*/*I* ratio is variable, and it decreases with age: age 20-24 years, 1.17; 25-29, 1.15; 30-34, 1.13; 35-39, 1.12; 40-44, 1.10; 45-49, 1.08; 50-54, 1.07; 55-59, 1.06; 60-64, 1.04; 65-69, 1.03; and 70-75, 1.02 [24].

30 : 15 ratio assesses HR response to standing up and consists of an initial increase in HR followed by a decrease in HR. The patient, after remaining for 3 minutes in a relaxed supine position, should quickly adopt the standing position in less than 3 seconds. The ratio of the R-R interval recorded at beat 30 and the R-R interval recorded at beat 15 after taking the standing position is calculated. The 30 : 15 ratio is variable and depends on the patient's age: age 20-29 years, 1.12; 30-39, 1.10; 40-49, 1.08; 50-60, 1.07; and 61-65, 1.06 [24].

Valsalva ratio assesses HR variability during an increase in intrathoracic pressure using the Valsalva maneuver. The Valsalva maneuver is influenced by both, the sympathetic system and the parasympathetic system [10]. When the patient is seated, he/she has to blow through a mouthpiece for 15 seconds, keeping the glottis closed constantly and continuously, maintaining a resistance of approximately 40 mmHg. The ratio between the longest R-R interval after the maneuver and the shortest R-R interval recorded during the 15 seconds of forced expiration is calculated. The normal ratio of the longest to the shortest R-R [24, 25] is also variable and decreases with age: age 20-29 years, 1.21; 30-39, 1.19; 40-49, 1.18; 50-60, 1.17; and 61-65, 1.16.

#### 2.4.1. Variation of SBP during Postural Changes

This test assesses sympathetic function and is performed in the same maneuver (positional change of supine position to standing) as the 30/15 ratio, and they are calculated jointly. The patient has to remain in the supine position for 3 minutes, during which blood pressure is measured 3 times. Afterwards, the patient has to adopt the standing position quickly (in less than 3 seconds). After that, 2 blood pressure measurements are taken. The difference between the mean of the SBP in decubitus and the average of the SBP in standing position is calculated. Normal response is a fall of SBP of <10 mmHg, borderline is a fall of 10-29 mmHg, and abnormal is a fall of >30 mmHg with symptoms [24].

CARTs were performed with *Neurotester*® (Italy, 2009). All the previous test values were transferred and processed by a computer program synchronized with *Neurotester*®. The normality values were adjusted according to the patient's age. The result of each test will be as follows: normal, borderline (or prephatological), or pathological. According to the *American Diabetes Association* (ADA) and the *American Academy of Neurology* recommendations, at least 2 abnormal HR tests are required for a confirmed diagnosis of CAN [3, 14, 26, 27].

### 2.5. Data Analysis

Continuous variables were described using mean and interquartile range for baseline and follow-up patient characteristics, and the mean and standard deviation for CARTs. Categorical values were summarized as proportions.

With respect to the statistical analysis, a paired two-tailed *t*-test was used for comparing results over time within each group for continuous variables. The Pearson test was used for categorical variables. When the available observations were limited, the Fischer exact test was used.

The level of significance was *p* < 0.05 for all the tests. Statistical analyses were performed using SPSS Statistics® (version 20). The Benjamini-Hochberg Procedure [28] correction was applied to minimize the likelihood of type 1 errors in multiple testing (results were described before and after the adjustment) with a false discovery rate of 10%.

### 2.6. Ethical Approval

This study was approved by *Comité Ético de Investigacion Clínica* (CEIC) of La Fe Health Research Institute, Valencia (Spain). This article contains human studies approved by CEIC. Written informed consent was obtained from all patients prior to their inclusion in the study.

## 3. Results

This study included 42 patients with T1DM (25 males and 17 females, with a mean age of 37.6 ± 5.6 years, mean BMI 23.9 ± 3.1 kg/m^2^, and a mean duration of diabetes 25.2 ± 6.2 years) who had undergone SPK transplantation and maintained functioning pancreatic and renal grafts during the follow-up. All patients were dialysis- and insulin-free and normoglycemic (their glycosylated hemoglobin levels were normal) at the moment of this study. Baseline and post-SPK follow-up characteristics are included in Table 1. Paired tests confirmed a significant improvement, especially in metabolic endpoints.

All patients achieved good control of arterial pressure, both at the time of SPK and during the follow-up, with or without antihypertensive treatment. Other known causes that could contribute to neuropathy were discarded in patients both at the time of SPK transplantation and during the follow-up.

Standard CARTs were used for the evaluation of autonomic neuropathy, on 31 patients before SPK transplantation, 16 of them again after 3 years, 13 after 5 years, 6 after 7 years, and 3 after 10 years.

**Figure 2:**
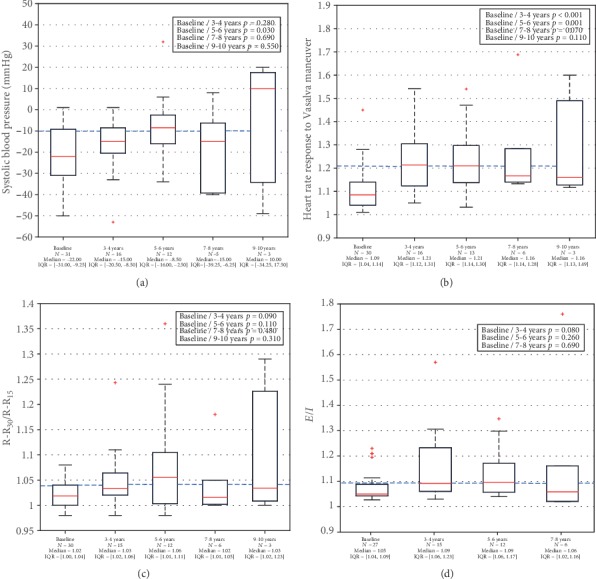
Results of cardiovascular function tests in SPK recipients, before and during the follow-up (textbox include paired *t*-tests). (a) Systolic blood pressure response to orthostasis. (b) Heart rate response to the Valsalva maneuver. (c) Heart rate response to orthostasis (30 : 15 ratio). (d) Heart rate variability with deep breathing (*E*/*I*). Data shown as median [interquartile range]. Interrupted line indicates lower limit of normal and borderline values from Sundkvist and Lilja (1985) [21] and Ewing et al. (1985) [23]. SPK: simultaneous pancreas and kidney transplantation.

Before the transplant procedure, all tests of autonomic function revealed abnormal results (Figure 2), obtaining a high prevalence of CAN: 96.8% (Table 1). After SPK transplantation, prevalence of CAN was progressively reduced over time (Table 1). Also, after SPK transplantation, paired patient sample analysis showed an improvement of SBP-response to orthostasis at the 5^th^ year after SPK (*p* = 0.03), as well as improvement of the Vasalva ratio at the 3^rd^ year (*p* < 0.001) and the 5^th^ year (*p* = 0.001) (Table 2).

After applying the Benjamini-Hochberg correction to multiple testing, the SBP response maintained significance after the 7^th^ year, and the Vasalva ratio also maintained a significant improvement after the 7^th^ year and after SPK. In addition, the 30 : 15 and *E*/*I* ratios showed significant improvement after the 3^rd^ year of the SPK transplantation (Table 3).

## 4. Discussion

CAN is a common complication of long-standing diabetes mellitus. Its etiopathogenesis is multifactorial, but hyperglycemia seems to be the primary cause, just as diabetic nephropathy. Several studies have shown that the longer the time of evolution of poorly controlled DM, the greater the complications. Long-term treatment of patients with short-duration of T1DM with intensive insulin therapy achieving normoglycemia leads to a reduced CAN prevalence as well and incidence. In fact, the *Diabetes Control and Complication Trial* (DCCT) compared an intensive therapy group versus conventional therapy, and showed that, although CAN prevalence increased in both groups, the incidence was significantly lower in the intensively treated group (28.9% vs 35.2%, *p* < 0.018) [10]. Also, it should be noted that intensive insulin therapy increases the risk of hypoglycemia, which, in turn, worsens CAN [29]. Furthermore, long periods of normoglycemia are required to retard CAN progression, and besides, this control is complicated using exogenous insulin. For these reasons, pancreas replacement seems to be the most logical treatment to restore the normoglycemic state in patients with T1DM and related end-stage kidney disease, who are candidates for kidney transplantation.

According to the inclusion criteria, patients undergoing SPK transplantation present terminal diabetic nephropathy that requires dialysis programs prior to transplantation. The pathogenic mechanisms that lead to diabetic nephropathy are the same that may produce diabetic retinopathy and neuropathy. Therefore, these patients would be expected to present retinopathy and diabetic neuropathy. In fact, diabetic neuropathy is very common and severe among candidates for SPK transplantation [7].

In our study, at baseline, all patients presented abnormal results in all CARTs. Mean time of evolution of T1DM was 25 years, and glycemic control was not optimal, according to ADA recommendations, with an HbA1C mean: 8.1% [14]. Therefore, it would be expected to find microvascular complications derived from DM such as CAN in this group of patients.

After pancreas transplantation, a slight improvement in cardiovascular autonomic function has been demonstrated in previous studies [18, 19]. Some of them reported that improvement of CAN seems to be slower and more progressive than the improvement of the peripheral neuropathy. However, Hathaway et al. [30] reported data from 23 recipients undergoing SPK and compared them with 16 patients undergoing kidney transplantation alone (KTA) followed for 12 months, and showed an improvement in autonomic nervous function already 1 year after transplantation for both groups of transplant recipients.

Our prospective 10-year study shows that cardiovascular autonomic function improves significantly after SPK transplantation. CARTs were analyzed individually using aged-stratified levels, as reflected in the Methods section. However, in Figure 2, normal values were considered according to Sundkvist and Lilja [21] and Ewing et al. (1985) [23], and reflected as an interrupted line, in order to facilitate the reading of the figure.

The patient population was analyzed using descriptive statistics and paired tests. Mean values of the observed variables (analytical and functional tests) for each time period were compared with the preoperative values (baseline).

Paired patient sample analysis showed that the Valsalva ratio and SBP response to orthostasis improved statistically at the 3^th^ and 5^th^ year after SPK, respectively. The *E*/*I* ratio and the 30 : 15 ratio did not reach significance for paired patient samples.

To reduce the chances of false discoveries in this multiple testing, the Benjamini-Hochberg correction [28] was applied to the results of the paired tests. After SPK transplantation and assuming a 10% of false discovery rate, the corrected tests showed an improvement of all cardiovascular function tests, but at different time points. The tests which showed an earlier improvement were Valsalva and 30 : 15 ratios and *E*/*I* ratio, as statistically significant differences were already found 3 years after SPK. However, these differences were maintained until 10 years after SPK only in the Valsalva ratio, and the SBP-response to orthostasis only reached statistical significance 5 years and 7 years post-SPK.

In the early stages, CAN may be completely asymptomatic and is detected only by a decrease in HR variability, subsequently leading to a clinical form characterized by resting tachycardia and exercise intolerance. In the most severe cases, it is accompanied by orthostatic hypotension [24, 26]. SPB response to orthostasis is, therefore, likely to be the last test to improve after SPK transplantation.

Regarding the sample size, in the basal period the interquartile range is lower than in the later periods, probably due to a larger sample size. However, attending SPS response to orthostasis, the interquartile range is higher in the basal period than in the 3r^d^-4t^h^ and 5^th^-6^th^ year after SPK. This could be the reason why SBP-response to orthostasis does not reach statistical significance 3 years after transplantation.

In all tests evaluated, the number of observations made in the 7^th^-8^th^ and 9^th^-10^th^ periods are not enough to draw a conclusion; hence, the great dispersion of the data is obtained.

Additionally, the authors have previously considered the development models for the assessment of the risk of SPK transplantation [31], in which predictors were selected on the basis of their contribution to the outcome, and subjects were grouped using supervised and unsupervised classification. Nevertheless, due to the hierarchical nature of the data collected in this study, future work will focus on the development of generalized linear mixed models (aggregating data point for each time interval and considering random effects).

Also, uremia and calcineurin inhibitors (CNI) as causes of neuropathy should be considered. Both diabetes and uremia contribute to the nerve dysfunction seen in uremic diabetic patients. After SPK transplantation, an initial improvement of neuropathy was probably due to the elimination of uremia. Subsequently, an additional improvement of the neuropathy was observed, which could be due to the repair and regeneration of the nerves. This late improvement was not seen in diabetic patients who had only undergone KTA. In our study, all patients underwent SPK transplantation. Uremia levels were high at the moment of SPK but decreased after SPK (Table 1).

CNI produce an abnormal nerve function, specifically reflecting nerve membrane depolarization [32]. For this reason, neuropathy is a highly prevalent complication in patients receiving CNI treatment. However, all our patients were treated with the same maintenance immunosuppression schedule, with no differences between them with regard to immunosuppression.

On the other hand, a meta-analysis of 15 longitudinal studies showed an association between CAN and increased mortality [3]. This risk has also been demonstrated by several studies. Silent myocardial ischemia is present in 20% of patients with CAN, compared to 10% in patients without CAN [33]. Furthermore, CAN is associated with a higher risk of peri- and intraoperative cardiovascular complications [5, 6, 12]. This could be due to the fact that CAN predisposes to life-threatening arrhythmias and sudden death [34] and is also associated with ventricular dysfunction [6, 35]. In this sense, the *Detection of Ischemia in Asymptomatic Diabetic Subjects* (DIAD) study showed that a decrease of the Valsalva ratio was associated with silent myocardial ischemia [36]. Also, CAN predicts the progression of diabetic nephropathy [37].

Consequently, CAN is considered an independent risk factor for mortality [37] and the strongest risk factor for all causes of mortality [3, 38–40].

CAN treatment is generally focused on alleviating symptoms [14]. But nevertheless, if the long-term normoglycemia induced by SPK transplantation can improve the CART results, SPK transplantation would reduce mortality and improve the prognosis of diabetic patients with CAN. Prospective studies would be needed to confirm this assumption.

This study has two main limitations. The first one is the limited sample size. If the sample size was larger, statistically significant results would probably be achieved in all evaluated periods. Despite this, the differences in CART results were enough to achieve statistical significance with nonparametric tests in some time periods. The second one is that, in our study, we did not follow a control group of diabetic patients not treated with SPK transplantation such as diabetic patients treated only with insulin therapy or diabetic patients undergoing KTA. With a control group of diabetic patients treated only with KTA, the effect of uremia on neuropathy could have been evaluated.

In conclusion, the prevalence of cardiovascular autonomic neuropathy in candidates for SPK transplantation is high and is generally advanced. Successful SPK transplantation normalizes blood glucose, glycosylated hemoglobin, and uremic levels, and improves CAN. Valsalva ratio is the most precocious test. Studies with a larger sample size would be required to confirm our data.

## Figures and Tables

**Table 1:** Paired tests for the baseline and post-SPK follow-up characteristics in SPK recipients.

Parameter	Baseline	Follow-up			
		3 years	5 years	7 years	10 years
*N*	42	31	16	7	4

BMI (kg/m^2^)	23.2 (21.9-25.5)	23.2 (20.9-25.7)	23.5 (22.5-26.5)	24.5 (22.2-26.1)	22.15(21.7-23.1)

Hemoglobin A1c (%)	7.8 (7.0-9.1)	5.5 (5.3-5.9)^†^	5.4 (5.1-5.5)^†^	5.7(5.1-5.9)^¤^	5.7 (5.1-5.9)

Basal glycemia (mg/dL)	168.5 (125.7-237.7)	86.0 (80.0-96.0)^†^	85.5 (80.0-94.7)^¤^	86.0 (75.0-98.0)^†^	83.0 (76.2-109.2)

C-peptide (ng/mL)	0.1 (0.1-0.4)	2.4 (1.8-3.2)^†^	2.2 (1.6-2.6)^†^	1.7 (1.0-2.1)^#^	1.6 (0.6-2.9)

S-creatinine (mg/dL)	7.1 (5.6-8.1)	1.1 (1.0-1.4)^†^	1.1 (1.0-1.3)^†^	0.9 (0.8-1.2)^#^	0.9 (0.9-1.0)^#^

Urea (mg/dL)	103.0 (84.0-138.0)	55.5 (46.5-63.2)^†^	49.0 (44.0-61.0)^†^	45.0 (39.0-68.0)^†^	39.0 (38.0-45.2)^#^

eGFR (mL/min/1.73m^2^)	11.5 (9.0-60.0)	72.0 (57.2-84.2)^†^	70.0 (53.5-85.5)^†^	85.0 (57.0-108.0)^†^	89.0 (75.2-99.0)^†^

Cholesterol (mg/dL)	156.5 (124.2-181.7)	164.0 (151.0-175.0)	178.0 (161.2-189.0)	158.0 (146.0-174.0)	169.5 (145.0-178.2)

HDL-C (mg/dL)	43.0 (34.5-59.5)	56.5 (45.2-70.0)	68.0 (58.5-75.7)	64.0 (56.0-69.0)	61.0 (61.0-.63.0)

LDL-C (mg/dL)	80.0 (51.0-97.5)	93.0 (84.0-104.0)	93.5 (79.5-103.7)	84.0 (76.0-92.0)	105.0 (78.0-106.0)

Triglycerides (mg/dL)	113.5 (86.2-167.0)	74.5 (55.0-87.7)^†^	67.0 (55.0-81.0)^†^	57.0 (46.0-71.0)	68.5 (55.5-76.2)

Systolic blood pressure (mmHg)	141.0 (130.0-156.0)	120.0 (113.7-130.0)^†^	128.0 (115.0-131.0)^#^	128.0 (105.2-130.0)	127.5 (113.7-139.7)

Diastolic blood pressure (mmHg)	76.0 (70.0-81.0)	70.0 (65.0-75.0)^¤^	70.0 (70.0-77.0)	71.0 (52.7-80.2)	75.5 (65.2-79.0)

Patients with hypolipemiant treatment	26 (61.9%)	8 (19.0%)^#^	5 (11,9%)^#^	1 (2.4%)^nd^	1 (2.4%)^nd^

Patients with antihypertensive treatment	40 (95.2%)	16 (38.1%)^#^	10 (23.8%)^#^	4 (9.5%)^†^	3 (7.1%)^†^

Patients with beta-blockers treatment	13 (30.9%)	3 (9.7%)^†^	1 (6.25%)^†^	0 (0%)^†^	0 (0%)^†^

Patients with diagnosis of CAN/patient with CARTs performed	30/31 (96.8%)	16/16 (100%)	11/13 (84.6%)	5/6 (83.3%)	2/3 (66.7%)

SPK: simultaneous pancreas and kidney transplantation; BMI: body mass index; eGFR: estimated Glomerular Filtration Rate; HDL-C: high-density lipoprotein cholesterol; LDL-C: low-density lipoprotein cholesterol; CAN: cardiovascular autonomic neuropathy. ^†^*p* < 0.001, patients at 3, 5, 7, or 10 years after SPK vs baseline. ^#^*p* < 0.01, patients at 3, 5, 7, or 10 years after SPK vs baseline. ^¤^*p* < 0.05, patients at 3, 5, 7, or 10 years after SPK vs baseline. nd: test not done.

**Table 2:** Results of cardiovascular autonomic tests, comparing evolution before and after SPK transplantation in paired patient samples. Statistically results (*p* < 0.05) are marked in bold.

Test	Comparison periods	Statistics				Correlations		Test	
		*N*	Mean	Standard Deviat.	Mean typical error	Correlation	Sig.	*t*	Sig. (bilateral)
SBP	Before SPK	17	-23.76	17.44	4.23	0.11	0.68	-1.13	0.28
	3^rd^ year		-18.00	13.83	3.35				
	Before SPK	16	-24.62	17.32	4.33	0.52	0.04	-2.36	**0.03**
	5^th^-6^th^ year		-15.66	12.81	3.20				
	Before SPK	4	-13.50	8.85	4.42	-0.89	0.11	0.45	0.69
	7^th^-8^th^ year		-20.50	23.27	11.64				
	Before SPK	3	-31.33	34.43	19.89	-0.89	0.30	-0.71	0.55
	9^th^-10^th^ year		0.33	45.28	26.14				

Valsalva ratio	Before SPK	17	1.11	0.12	0.03	0.49	0.05	-3.46	**0.00**
	3^rd^ year		1.21	0.13	0.03				
	Before SPK	17	1.09	0.07	0.02	0.52	0.03	-5.39	**0.00**
	5^th^-6^th^ year		1.23	0.13	0.03				
	Before SPK	5	1.12	0.08	0.04	0.92	0.03	-2.41	0.07
	7^th^-8^th^ year		1.29	0.23	0.10				
	Before SPK	3	1.13	0.11	0.06	0.87	0.33	-2.79	0.11
	9^th^-10^th^ year		1.36	0.22	0.13				

30 : 15 ratio	Before SPK	16	1.02	0.03	0.01	0.35	0.18	-1.83	0.09
	3^rd^ year		1.05	0.06	0.02				
	Before SPK	16	1.03	0.05	0.01	0.05	0.85	-1.72	0.11
	5^th^-6^th^ year		1.10	0.15	0.04				
	Before SPK	5	1.01	0.02	0.01	-0.72	0.17	-0.78	0.48
	7^th^-8^th^ year		1.05	0.08	0.03				
	Before SPK	3	0.99	0.01	0.00	-0.84	0.36	-1.34	0.31
	9^th^-10^th^ year		1.11	0.15	0.09				

*E*/*I* ratio	Before SPK	11	1.10	0.08	0.02	0.77	0.00	-1.98	0.08
	3^rd^ year		1.05	0.06	0.02				
	Before SPK	12	1.08	0.07	0.02	0.70	0.01	-1.20	0.26
	5^th^-6^th^ year		1.11	0.11	0.03				
	Before SPK	2	1.06	0.03	0.02	1.00	0.00	0.52	0.69
	7^th^-8^th^ year		1.06	0.02	0.01				

SBP: systolic blood pressure response to orthostasis; 30 : 15 ratio: heart rate response to orthostasis; *E*/*I* ratio: expiration/inspiration or deep breathing ratio; SPK: simultaneous pancreas kidney transplantation.

**Table 3:** Benjamini-Hochberg correction for multiple testing in autonomic tests.

Variable	Baseline compared to	*p* value of paired test	Rank	*α* (*k*/*m*)^1^
Valsalva ratio	5^th-^6^th^ year	0.00001	1	0.0067
Valsalva ratio	3^th-^4^th^ year	0.001	2	0.0133
Systolic blood pressure response to orthostasis	5^th-^6^th^ year	0.03	3	0.0200
Systolic blood pressure response to orthostasis	7^th-^8^th^ year	0.069	4	0.0267
Valsalva ratio	7^th-^8^th^ year	0.07	5	0.0333
*E*/*I* ratio	3^th-^4^th^ year	0.08	6	0.0400
**30 : 15 ratio**	**3** ^**th-**^ **4** ^**th**^ ** year**	**0.09**	**7**	**0.0467**
30 : 15 ratio	5^th-^6^th^ year	0.11	8	0.0533
Valsalva ratio	9^th-^10^th^ year	0.11	9	0.0600
*E*/*I* ratio	5^th-^6^th^ year	0.26	10	0.0667
Systolic blood pressure response to orthostasis	3^th-^4^th^ year	0.28	11	0.0733
30 : 15 ratio	9^th-^10^th^ year	0.31	12	0.0800
30 : 15 ratio	7^th-^8^th^ year	0.48	13	0.0867
Systolic blood pressure response to orthostasis	9^th-^10^th^ year	0.55	14	0.0933
*E*/*I* ratio	7^th-^8^th^ year	0.69	15	0.1000

^1^ *α*: false discovery rate. Estimated at 10%. *k*: rank. *m*: total number of tests (15).

## Data Availability

Maria Argente-Pla and Juan Francisco Merino-Torres are the guarantors of this work and, as such, had full access to all the data in the study and take responsibility for the integrity of the data and the accuracy of the data analysis. The data used to support the findings of this study are available from the corresponding author upon reasonable request.
